# A Determination of Salivary and Serum Glucose Levels in Patients With Type II Diabetes Mellitus

**DOI:** 10.7759/cureus.54395

**Published:** 2024-02-18

**Authors:** Bushra Anjum, Neeharika Soorneedi, J Swathi, Mohammed Imran, Kavitha Gaddikeri, Anoop Nallapu

**Affiliations:** 1 Department of Oral Pathology and Microbiology, Panineeya Institute of Dental Sciences and Research Centre, Hyderabad, IND; 2 Department of Oral Pathology and Microbiology, Malla Reddy Institute of Dental Sciences, Hyderabad, IND; 3 Department of Oral Pathology and Microbiology, Government Medical College and Hospital, Hyderabad, IND; 4 Department of Oral Pathology and Microbiology, Sri Sai College of Dental Surgery and Hospital, Hyderabad, IND; 5 Department of Oral Pathology and Microbiology, Employees' State Insurance Corporation Dental College and Hospital, Gulbarga, IND; 6 Department of Healthcare Administration and Service Management, Conestoga College, Brantford Campus, Brantford, CAN

**Keywords:** glucose levels, diabetes mellitus type 2, saliva, serum, salivary alpha amylase

## Abstract

Aim: The aim of this work was to determine and compare serum and salivary glucose levels in patients with type II diabetes mellitus (DM).

Material and methods: This study had an overall sample size of 100, which consisted of male and female volunteers aged 30-67 years. Both in-depth interviews and physical tests were conducted. Blood and saliva were collected from the participants while fasting, which were analyzed to determine the levels of salivary alpha-amylase (α-amylase). Both the subjects and the controls were instructed to test their blood glucose levels while fasting and it was suggested that HbA1c values will be used for diagnosing diabetes following the guidelines of the American Diabetes Association, Centers for Disease Control, and World Health Organization.

Results: The average age of the control group (Category A) was noted as 47.52±6.28 years, and that of the study group (Category B) was 49.17±7.25 years. In Category A, female (n=23) were 46%, and 54% were male (n=27); and 40% of the people in Category B were female (n=20), and 60% were male (n=30). The majority of patients (54%) in Category B displayed an average level of DM control (n=27), followed by poor control (24%. n=12), well-controlled (20%, n=10), and uncontrolled DM (2%, n=1). Category A had an average salivary α-amylase concentration of 3.1±0.88 U/L, whereas that of Category B was 12.06±2.36 U/L. Thus, the mean salivary α-amylase level of Category B was found to be much higher than that of Category A, and this difference was statistically significant (p<0.001).

Conclusion: The determination of α-amylase levels in the saliva of individuals suspected of having type II DM has been suggested as a potential diagnostic method. Screenings conducted at healthcare institutions and community health fairs, as well as epidemiological studies, might benefit from this method. We believe that normal clinical practice should include the use of saliva in a broad variety of diagnostic tests.

## Introduction

Diabetes mellitus (DM) is a metabolic disorder marked by elevated blood glucose levels, known as hyperglycemia, and disruption of the metabolism of carbohydrates, proteins, and lipids [1]. There are two recognized triggers for diabetes, which are denoted as type 1 and type 2 diabetes. In type 1 diabetes, host immune cells (i.e., T lymphocytes) attack the pancreatic beta cells and destroy the insulin-secreting pancreatic beta cells, causing the pancreas to stop insulin secretion further [2]. With decreased levels or no insulin in the blood, the glucose fails to enter the cells, leading to glucose build-up in the bloodstream (i.e., hyperglycemia). In contrast, in type 2 diabetes, peripheral target organs become resistant to the effects of insulin (i.e., insulin resistance builds up in the patient's body) [3]. The number of individuals with diabetes is expected to rise to 320 million by 2025 [4,5]. Currently, blood glucose testing (serum) is the only reliable method for diagnosing diabetes. People experience both physical and emotional discomfort during this testing, which often involves a venipuncture or a finger stick. As such, it would be preferable to have a process that is easy, painless, and non-invasive, such as measuring the amount of glucose in saliva [6]. Studies have revealed that a person's hormonal, immunologic, neurologic, nutritional, and metabolic health affects the composition of the saliva, thus making this biological fluid an immensely valuable indicator of both local and systemic alterations [7-10]. Individuals with persistently elevated blood glucose levels are at greater risk of periodontal disease, candidiasis, and dental caries [8]. Evidence suggests that people with diabetes have greater levels of glucose in their saliva than those without diabetes [9]. However, there is inconsistency in the reporting of how blood glucose levels compare to those in saliva [10]. These discrepancies may result from using different populations as samples, or from variations in collecting saliva or performing glucose tests [11]. Many factors, including oral retention of ingested carbohydrates and glucose utilization by oral bacteria, may contribute to the poor correlation between blood and saliva glucose concentrations commonly observed in diabetic patients [12].

The timely and early diagnosis of DM is very important, as it is considered as a silent killer. Misdiagnosis is also possible, as the disease may not show any symptoms initially, resulting in increased rates of morbidity and mortality [13]. This association emphasizes the necessity of a prompt diagnosis, which would aid in the successful management of the patient’s illness [14]. For this monitoring, susceptible individuals must undergo frequent blood draws, which is often a cumbersome procedure causing great discomfort. Therefore, there has been a recent upsurge in curiosity about non-invasive diagnostic approaches for DM, including the collection of extra-physiological fluids (e.g., saliva), as substances expressed in serum may also be secreted in the saliva [15].

Saliva is an organic bio-fluid that may be used to detect the presence or absence of disease in a person; it can also be used as a medium for monitoring the health of an individual or population [16]. As saliva can serve as a sign of systemic and local abnormalities that accompany illness, it is conceivable to employ it as a diagnostic medium containing the same components as serum [17]. Many studies have connected DM to alterations in salivary gland function and salivary composition, including protein levels and salivary enzymes [18]. For example, DM affects the levels of both cyclic adenosine monophosphate (cAMP) receptors and alpha-amylase (α-amylase). Salivary α-amylase, present in saliva as an important salivary protein, is secreted as a digestive metalloenzyme in the acinar cells of the salivary glands. DM causes changes in certain salivary components (e.g., α-amylase and cAMP receptors), leading to altered function of salivary glands [17,18]. These alterations in the protein secretion, in conjunction with increased permeability of the basement of salivary glands, contribute to an increased expression of salivary α-amylase. Based on this biological alteration, the current study attempts to estimate salivary α-amylase and its expression in the saliva of DM patients, with the goal of using salivary α-amylase as a potential diagnostic indicator of type 2 DM.

## Materials and methods

A total of 100 individuals comprising both male and female patients ranging in age from 30 to 67 years old who were attending the Department of Oral Medicine and Oral Pathology, Bibi Amena Dental Hospital & Implant Centre, Chandrayangutta, Hyderabad, Telangana, India, were enrolled in this study after obtaining their relevant medical history and history of diabetes. The study was carried out from April 2023 to June 2023 and aimed to determine salivary and serum glucose levels in patients with type 2 DM. The study protocol underwent complete assessment and received approval from the ethical committee at Bibi Amena Dental Hospital and Implant Centre (BAMDH/2023/EC29). The study was explained to the patient in their own language to facilitate completion and comprehension of questions. Before the commencement of the study, patient informed consent was obtained in their own native language. Both the study subjects and the controls were given the instruction to test their blood glucose levels while they were fasting to determine blood glucose levels HbA1c (glycated hemoglobin) values, which will be applied for diagnosing diabetes as recommended by the American Diabetes Association, Centers for Disease Control, and World Health Organization. They have suggested that HbA1c is a possible indicator for the determination of glucose levels in diabetic patients, as HbA1c level is an independent risk factor for cardiac disorders. For non-diabetic patients, HbA1c falls within the 4.0-5.6% range, that of prediabetes commonly falls within 5.7-6.4%, and those having an HbA1c of ≥6.5% are considered diabetic. Fasting blood and saliva samples were collected from both the control group (Category A) and the study subjects (Category B), which were later analyzed to evaluate the amount of α-salivary amylase present.

Sample size determination

The sample size was determined based on a pilot study of 10 subjects. Based on 5% margins of error (degree of error in results obtained during random sampling method) and 95% confidence intervals, the estimated sample size was determined to be 50 in each Category, leading to a total sample size of 100.

Data collection

In the control group (Category A), 2 mL of blood was drawn from the antecubital vein while fasting under sterile conditions and subsequently labeled. This sample was centrifuged for 15 min at 3000 rpm, after which the serum obtained was collected and transferred to a plastic fluoride oxalate tube to determine the quantity of glucose in the blood. The fluorides and oxalates in the tube act as weak anticoagulants that are used to preserve the blood and help to prevent glycolysis; the application of such substances helps to determine the levels of glucose and HbA1c in the blood.

In the study group (Category B), 2 mL of blood was obtained from the antecubital vein while fasting under sterile conditions and subsequently labeled following the protocol that was followed for Category A. Each sample was centrifuged for 15 min at 3000 rpm, and the serum was collected and transferred to a plastic fluoride oxalate tube.

Sialo chemical procedure

The participants of both categories (category A and category B) were given instructions to gargle with water for a considerable amount of time. Later they were instructed to spit the saliva into clean containers whenever saliva spontaneously accumulated in their mouth, which allowed for the collection of saliva samples. A total of 2 mL of saliva was collected and stored in an ice pack. Saliva samples stored in ice packs or containers at temperatures between 2°C and 8°C will remain stable for up to 24 hrs, preventing any bacterial growth or degradation of salivary molecules while being transported for further laboratory processing. After the samples were collected, they were dispatched to the laboratory and centrifuged for 15 min at a speed of 4000 rpm in order to separate the supernatant from the residual saliva. In the last phase of this investigation, an enzymatic colorimetric test was carried out to determine the salivary α-amylase concentration presence in the saliva.

The inclusion criteria for this study encompassed persons who were considered healthy without any abnormalities in their blood sugar levels. Individuals diagnosed with type II diabetes mellitus were also included.

Conversely, the exclusion criteria comprised individuals receiving therapy for disorders affecting the salivary glands or those who had undergone salivary gland surgery. Additionally, patients who had cancer of the head and neck, and had received radiation treatment or chemotherapy, were excluded. Finally, patients with type II diabetes mellitus, along with any other systemic disorders or unhealthy behaviors, were not included in the study.

Statistical analysis

The salivary α-amylase data acquired was analyzed using Student’s t-test and the receiver operating characteristic (ROC) curve was established.

## Results

**Figure 1:**
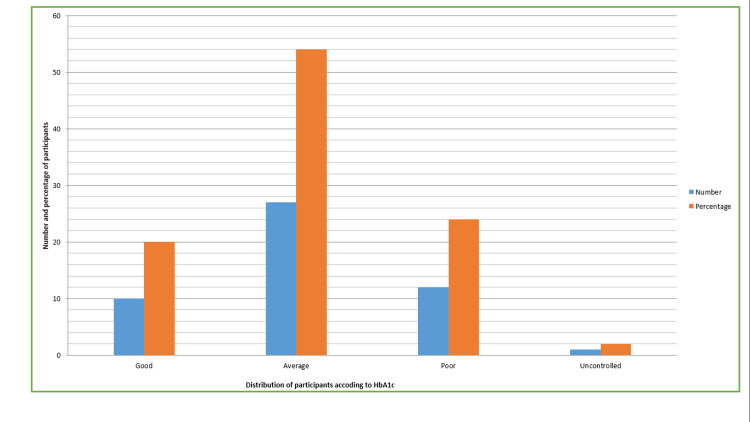
Distribution of participants in Category B according to HbA1c (glycated hemoglobin) The figure provides the comparative analysis of the distribution of participants in Category B according to HbA1c values (glycated hemoglobin). The majority of the patients (54%) in category B displayed an average level of DM control (n=27), followed by poor control (24%, n=12), well-controlled (20%, n=10), and uncontrolled DM (2%, n=1).

A total of 100 individuals were chosen as participants for the study, ranging in age from 30 to 67 years, including people of both sexes. The average age of the participants in Category A was noted as 47.52±6.28 years, and that of Category B was 49.17±7.25 years. In Category A females (n=23) were 46%, and 54% were males (n=27); and 40% of the people in Category B were females (n=20), and 60% were males (n=30) (Table 1). The majority of patients (54%) in category B displayed an average level of DM control (n=27), followed by poor control (24%. n=12), well-controlled (20%, n=10), and uncontrolled DM (2%, n=1) (Table 2, Figure 1). In Category A an average salivary α-amylase concentration of 3.1±0.88 U/L was observed, whereas in Category B the concentration observed was 12.06±2.36 U/L. When compared to Category A, the mean salivary α-amylase level in Category B was noted to be much higher (Table 3), and this difference was statistically significant (p<0.001). The ROC curve generated from the data showed a cut-off value of <5 U/L for detecting type 2 DM via salivary α-amylase (Table 4), with a specificity of 100% and a sensitivity of 95%.

**Table 1:** Gender and age distribution in Category A and Category B Data is represented in percentage (%) and frequency (n). The table presents the gender and age distribution in Category A and Category B. The average age of those who fell into Category A was noted as 47.52±6.28 years, and that of Category B was noted as 49.17±7.25 years. In Category A, females (n=23) were 46%, and 54% were males (n=27); and 40% of the people in Category B were females (n=20), and 60% were males (n=30).

Gender	Category A=50		Category B=50	
	Frequency	Percentage	Frequency	Percentage
Male	27	54	30	60
Female	23	46	20	40
Age in years				
below 40	12	24	10	20
40-50	30	60	25	50
50-60	5	10	10	20
Above 60	3	6	5	10
Mean Age	47.52±6.28		49.17±7.25	

**Table 2:** Distribution of participants in Category B according to HbA1c values (glycated hemoglobin) Data is represented in percentage (%) and frequency (n). The table provides a comparative analysis of the distribution of participants in Category B according to HbA1c values (glycated hemoglobin). The majority of patients (54%) in Category B displayed an average level of DM control (n=27), followed by poor control (24%. n=12), well-controlled (20%, n=10), and uncontrolled DM (2%, n=1).

	DM Control			
HbA1c value (g/dL)	Good (≤8%)	Average (>8–≤10%)	Poor (>10–≤12%)	Uncontrolled (>12%)
Number	10	27	12	1
Percentage	20	54	24	2

**Table 3:** Estimation of salivary α-amylase in Category A and Category B Data is represented in mean value. The table provides a comparative analysis estimation of salivary α-amylase in Category A and Category B. Category A had an average salivary α-amylase concentration of 3.1±0.88 U/L, whereas that of Category B was 12.06±2.36 U/L. When compared to Category A, the mean salivary α-amylase level in Category B was observed to be much higher and this difference was statistically significant (p<0.001).

	Category A (n=50)	Category B (n=50)	T value	P value
Salivary α-amylase			-11.29	0.001
Range (U/L)	1-5	5-20		
Mean value (U/L)	3.1±0.88	12.06±2.36		

**Table 4:** Receiver operating characteristic curve * In table indicates a statistically significant P-value. The table depicts the receiver operating characteristic (ROC) curve. The ROC curve generated from the data showed a cut-off value of <5 U/L for detecting type 2 DM via salivary α-amylase, with a specificity of 100% and a sensitivity of 95%.

Area Under the Curve	Standard Error	P-Value	95% Confidence Interval	
			Lower Bound	Upper Bound
0.98	0.0039	<0.001*	0.97	1.001

## Discussion

Saliva comprises a variety of different components, the most notable of which is water; other components include salt, potassium, glycoproteins, glucose, and amino acids. It has been shown that saliva is an efficient tool for diagnosing both local and systemic conditions [18]. Recent advances in diagnostic technology have greatly increased the likelihood of achieving the long-term goal of developing clinically validated saliva-based diagnostics for health monitoring and the early diagnosis of oral illness and other systemic disorders.

Both quality of life and life expectancy were negatively impacted by the microvascular and macrovascular consequences of chronic hyperglycemia [19]. As a result, early diagnosis is necessary to provide suitable treatment and avoid additional complications [20]. As saliva is a biological medium that has been termed the “mirror of the body,” it is a great choice for analyzing health and disease via certain indicators [21]. Salivary α-amylase is an enzyme produced by the salivary glands that is responsible for the hydrolysis of starch into dextrins and monosaccharides. An increase in these monosaccharides, each of which is composed of glucose units, is what causes hyperglycemia to occur [22]. This increase results in alterations to the proteins that are responsible for secretion, as well as an increase in the permeability of the salivary gland’s basement membrane. As a direct consequence of this alteration, α-amylase production is increased, which is then released into the saliva and oral cavity.

In the current study, the average age of those in Category A noted was 47.52±6.28 years, and that of Category B was 49.17±7.25 years. A similar mean age distribution was also observed in a study carried out by Malathi et al. [23]. Data suggest that diabetes often manifests in individuals at approximately the age of 40 and above.

With respect to gender, Category A comprised 46% women and 54% men, whereas Category B comprised 60% women and 40% men. These data support the conclusion drawn by Aregbesola et al. [24], who observed a gender difference in the prevalence of type 2 DM, with a higher prevalence observed in males, which may be due to the fact that the number of male participants was high in their study.

In our study, most of the DM patients were found to have their diabetes in a state of moderate control (54%), followed by poor control (24%) and good control (20%), and only 2% of patients exhibited a state of uncontrolled DM. These results might be because patients receiving treatment would have been taking their prescribed medicines regularly on time, as well as strictly following the instructions given to them related to food, nutrition, exercise, and mental health.

The saliva of healthy individuals does include glucose, but the mechanism that triggers the release of glucose into the saliva remains unknown; although it has not been proven beyond a reasonable doubt, the involvement of paracellular and intercellular pathways has been postulated. Many studies have attempted to explain the elevated amounts of glucose that are seen in the saliva of diabetic patients. Lopez et al. [25] provided evidence that the salivary glands operate as blood glucose filters that are either hormonally or neurally controlled. According to Qureshi et al. [26], persistent hyperglycemia leads to changes in the basement membrane of the salivary glands, as well as microvascular abnormalities in the blood vessels - both of which may be life-threatening. The concentration of glucose in saliva rises when there is an increase in the amount of glucose that escapes from the ductal cells of the salivary glands.

Sreedevi et al. [27] stated, referring to a study conducted by Powers [28], that glucose is a very small molecule that may easily permeate across semipermeable membranes. Therefore, when blood glucose levels are elevated, as they are in diabetes, a significant amount of glucose is made available to the saliva [29]. Changes in permeability brought on by modifications to the basement membrane induced by diabetes may potentially explain the greater concentration of glucose that is seen in the saliva of diabetic patients [30].

Alterations in the microvascular system are known to have a role in diabetic complications, which has led to several related theories [31]. Such complications include an increased risk of atherosclerosis, cerebrovascular, cardiovascular events, and early death. Micro blood vessels, which consist of arterioles, venules, and capillaries, are affected at both the cellular and architectural levels in diabetic patients, causing disruption of blood supply and nutrients at the microvascular level, leading to diabetic microangiopathy. These events lead to alteration in the basement membrane of blood vessels, which, in turn, causes dysfunction in the integrity of the vessel endothelium, leading to increased permeability (leaky blood vessels) [32]. Another complication is the increased production of advanced glycosylation end products, which causes disruption in their normal functioning due to altered molecular conformation, disrupted enzyme activity, and receptor dysfunction. These products accumulate in different cell types intra and extracellular with crosslinking of proteins, lipids, collagen, and other proteins leading to the complicated state of high blood glucose levels [33]. Other byproducts of prolonged hyperglycemia include sorbitol, diacylglycerol, and fructose-6-phosphate; these byproducts have an effect on the proteins of the extracellular matrix, which, in turn, has an effect on the basement membrane [34]. A leaky microvasculature and a dysfunctional basement membrane, which are both complications of DM, may be to blame for the condition’s increased glucose transfer from the blood into the saliva [29-32].

Category A had an average salivary α-amylase concentration of 3.1±0.88 U/L, whereas in Category B much higher level of 12.06±2.36 U/L was observed, and this difference was statistically significant (p<0.001). In those with DM, although blood glucose levels are increased, those in the interstitial tissue are decreased. The stimulation of the pancreatic islets of Langerhans, pancreatic acinar cells, and salivary gland acinar cells is caused by the activation of the glucose control system in the brain in response to the stimulus of reduced blood glucose levels [22].

The two categories exhibited considerably different median salivary α-amylase levels while fasting (2.5 U/L and 11.6 U/L for Category A and Category B, respectively). When compared to Category A, the average amount of salivary α-amylase in Category B was much greater. Thus, individuals with DM were found to have significantly higher salivary α-amylase levels than those of the controls, which lends credence to the notion that an increase in the amounts of α-amylase that are present in the saliva is associated with DM. This difference was highly statistically significant (p<0.001). This finding is in line with those of studies conducted by other authors [29-33]. According to two Indian investigations conducted by Indira et al. [34] and Panchbhai et al. [35], those with type II diabetes had lower salivary α-amylase levels than those who did not have the condition.

Alterations in the permeability of the basement membrane could potentially cause an increase in the amount of serum-derived components that leak into the saliva via gingival crevices [36]. People suffering from diabetes will have elevated levels of amylase and cAMP receptors in the annular sections of their salivary glands, which is caused by an increase in the permeability of the basement membrane of the salivary glands, making it possible for salivary proteins (e.g., amylase) to enter their mouths through their secretions and make their way into their mouths [37]. This notion adds support to the hypothesis that persons who have DM have higher amounts of α-amylase in their saliva than healthy individuals [31].

The ROC curve generated from our results demonstrated that salivary α-amylase had a sensitivity of 95% and a specificity of 100% in identifying type II DM. Consequently, a cut-off value of 5 U/L was determined to be appropriate. Thus, participants in this study with blood glucose levels of >5 U/L were considered to have DM.

This study did have some limitations. First, very viscous saliva could not be used for assessment, as it does not precisely reflect the concentration of chemicals in blood and hence cannot be employed. According to the findings of this study, saliva should be investigated further to determine its potential in diagnostic applications. Our capacity to detect DM at an earlier stage via saliva, which may then be utilized to prevent oral mucosal disorders and the following consequences of such diseases, represents the main contribution of this work. Saliva is a biological and diagnostic tool that is inexpensive, painless, and simple to collect; and does not involve any intrusive procedures, making it an effective, time-saving, affordable non-invasive diagnostic method.

## Conclusions

Saliva has been of particular interest to researchers due to the wide diversity of enzymes and compounds that it contains. In addition, the oral fluid contains chemical components that are identical to those present in the serum. Saliva has a number of distinct advantages, the most notable of which is that it can be gathered in a non-invasive manner by anybody, regardless of their level of education, and that it can be collected at a low cost in large quantities. The determination of α-amylase levels in the saliva of individuals suspected of having type 2 DM has been suggested as a potential diagnostic method. Screenings conducted at healthcare institutions, those conducted at community health fairs, and epidemiological studies might all benefit from this method. In conclusion, we believe that normal clinical practice should include the use of saliva in a broad variety of diagnostic tests.
